# Selecting a dietary assessment method for a national nutrition survey: a review and evaluation of online 24-h dietary recall tools

**DOI:** 10.1017/S1368980024002507

**Published:** 2024-12-05

**Authors:** Berit Follong, Sally Mackay, Cliona Ni Mhurchu

**Affiliations:** 1 National Institute for Health Innovation, The University of Auckland, Auckland 1023, New Zealand; 2 Department of Epidemiology and Biostatistics, School of Population Health, The University of Auckland, Auckland 1023, New Zealand

**Keywords:** Public health, 24HDR, Food intake, Technology, Web-based

## Abstract

**Objective:**

Online 24-h dietary recall tools are commonly used in nationwide nutrition surveys to assess population diets. With a steep rise in the development of new and more advanced 24-h dietary recall tools, the decision of which tool to use for a national nutrition survey becomes increasingly challenging. Therefore, this short communication outlines the process of selecting a 24-h dietary recall tool for a national nutrition survey in New Zealand.

**Design:**

To identify suitable 24-h dietary recall tools, a review of peer-reviewed and grey literature was conducted (2019–2022). Data on functionalities, validation, usability and adaptability were extracted for eighteen pre-specified tools, which were used in the subsequent evaluation process.

**Results:**

Six of the eighteen tools had new relevant publications since 2019. The fourteen new publications described six validation studies and eight usability studies. Based on pre-selection criteria (e.g. availability, adaptability, previous use in national surveys), three tools were shortlisted: ASA24, Intake24 and MyFood24. These tools were further evaluated, and expert advice was sought to determine the most suitable tool for use in the New Zealand context.

**Conclusions:**

A comprehensive yet time- and cost-efficient approach was undertaken to identify the potential use of online 24-h dietary recall tools for a national nutrition survey. The selection process included key evaluation criteria to determine the tools’ suitability for adaptation within the New Zealand context and ultimately to select a preferred tool. A similar approach may be useful for other countries when having to select 24-h dietary recall tools for use in national nutrition surveys.

National nutrition surveys collect population-based diet-related data that are essential in assessing food and nutrient intakes, monitoring nutritional status and informing and evaluating public health nutrition programmes and policies. Given the significance of the data obtained through these national surveys, it is important that the methodologies used are robust and valid^(1)^.

The most common dietary assessment method used in national nutrition surveys is the 24-h dietary recall^(2,3)^, due to its standardised data collection process and its ability to provide reasonably accurate nutrient intake data and collect data from large population groups while minimising participant burden. Many traditional 24-h dietary recall tools have undergone technological advancements in recent years to enhance their cost-efficiency, data quality, user experience and scalability^(4,5)^. Today, a wide range of online 24-h dietary recall tools are available, each varying in their functionalities, ease of use, validity and adaptability^(2,6)^. Selecting a 24-h dietary recall tool to collect population dietary intake data can therefore be challenging.

The methods for a future national nutrition survey in New Zealand were developed recently. Since the 24-h dietary recall tool used in the previous national nutrition surveys was no longer available, a new tool had to be selected. This short communication describes the process and results of a review and evaluation of online 24-h dietary recall tools for adaptation and use in a New Zealand national nutrition survey.

## Methods

### Updated review of existing tools

To identify existing online 24-h dietary recall tools that could potentially be adapted for use in a New Zealand nutrition survey, a systematic review published in 2021 by Gazan and colleagues was updated^(2)^. The original review aimed to provide an overview of online 24-h dietary recall tools that had the potential to collect dietary intake data for national nutrition surveys and therefore provided an ideal starting point. The authors identified eighteen online 24-h dietary recall tools in publications between 2000 and 2019. We decided to update Gazan’s review for the 24-h dietary recall tools already identified and therefore limited the updated searches to the same eighteen tools. Any tools developed after the initial search was performed would have been in an early stage of development, and it was therefore less likely that validation and usability studies would have been conducted. The selection of a well-established tool was preferred over a new tool that required more testing. Furthermore, the contractor (New Zealand Ministry of Health) advised that they would prefer a 24-h dietary recall tool that had already been used in nutrition surveys in other countries and tool selection needed to be completed within a short timeframe to meet contract deliverables.

#### Search strategy, study selection and data extraction

The search for relevant online 24-h dietary recall tools was updated (from 2019 to January 2022) using the same methods (i.e. search strategy and eligibility criteria) described in Gazan *et al.* ^(2)^. The original search terms were combined with the names of the eighteen 24-h dietary recall tools to identify relevant publications in PubMed and Google (Scholar).

Study selection was based on publications or (scientific) literature that described the functionality, validity, user usability or flexibility of relevant online self-administered 24-h dietary recall tools^(2)^. Relevant studies were only included if published in English. A standard screening process for rapid scoping reviews was performed^(7)^ to determine eligibility. Search results were initially screened for inclusion based on title and abstract by one researcher (BF) followed by assessment by a second independent reviewer (SM) of a random sample (∼20 %) of excluded records only. Full-text articles were obtained and screened following the same process. Records marked as ‘unsure’ or where decisions were in conflict were resolved through discussion by both researchers or with the wider research team.

Data extraction was undertaken in line with the previous review and included updated information on tools’ characteristics, collection methods and functionalities (e.g. dietary recall steps) and any validation or usability studies^(2)^. One researcher (BF) extracted and tabulated the data, which were subsequently checked by a second researcher (SM). Authors and/or owners of the recall tools were contacted with requests to provide missing data or verify information where needed.

### Evaluation

#### Shortlisting, in-depth evaluation and consultation

Evaluation of the online 24-h dietary recall tools was divided into three steps including creation of a shortlist, in-depth evaluation and expert consultation. Tools were scored against a set of pre-defined criteria to shortlist those with the highest potential for use in a New Zealand nutrition survey. These pre-selection criteria were based on criteria used for the UK National Diet and Nutrition Survey^(8)^ with additional criteria related to specific requirements for a New Zealand survey (Table 1). Using a stepwise approach, the first five criteria were scored (0, 1 or 2 points each) and subtotalled. Any tools that did not meet criteria 3 or 4 or scored ≤ 4 points for criteria 1 to 5 were eliminated. The remaining tools were scored for criteria 6–8 and shortlisted if total scores were > 7 points. This cut-off score was based on advice from an expert advisory group (including eighteen members with expertise in nutrition and public health) and aimed to ensure essential criteria were met before advancing to the next evaluation stage. The scoring was conducted by one researcher (BF) and checked by a second (SM). Both have a background in public health nutrition and dietary assessment methods.

**Table 1.** Pre-selection criteria used to shortlist 24-h dietary recall tools

#	Criteria	Scoring
1	Evidence of validation of 24-h dietary recall tool	0 points; no validation studies 1 point; 1–2 validation studies 2 points; 3 or more validation studies
2	Evidence of use in previous (national) surveys or large studies	0 points; only used in small or non-national studies/surveys 1 point; used in large studies only 2 points; used in national surveys
3	The tool is available for use	0 points; no 1 point; yes
4	The tool is in English or can easily be adapted to English	
5	The tool can use images to portray portion size	
6	The tool can record (or can be easily adapted to record) additional details required to accurately code dietary data (e.g. cooking methods, brands, different nutrient profiles of foods)	
7	The tool can be updated to align with the expected requirements for a New Zealand national nutrition survey (e.g. adding or changing language, portion size images, national food lists and nutritional composition data)	
8	The tool can automatically link food/drink consumption to food composition data	

Shortlisted tools were then assessed against a larger set of evaluation criteria from the UK National Diet and Nutrition Survey selection process^(8,9)^. Several criteria were added to determine the suitability of the tool for New Zealand by aligning with specific requirements for a national survey and priority ethnic groups. These added criteria were developed in consultation with expert advisors and key representatives of the Ministries of Health and Primary Industries. The final evaluation criteria were grouped into four categories: (1) organisational, logistical and financial aspects (e.g. ‘How many respondents can use the tool at the same time?’); (2) applicability (e.g. ‘Will the tool be suitable for the population groups of interest?’); (3) respondent, interviewer and research usability (e.g. ‘Can the tool be used offline?’); and (4) accuracy and precision (e.g. ‘Does the tool prompt for foods commonly consumed together?’). The full set of criteria can be provided upon request.

To evaluate the shortlisted 24-h dietary recall tools, the relevant details related to the criteria above were extracted from the updated scoping review. Additionally, relevant websites were searched, and tool developers or owners or corresponding authors of studies were contacted. The key strengths and limitations of each tool were identified.

In-person consultation with our nutrition and public health expert advisors was undertaken to guide the decision on which tool to select and adapt for use in a nutrition survey. Where needed, further information about the tools was collected to facilitate the discussions.

## Results

### Updated review of existing tools

After removing fifty-three duplicates, 644 records were screened for inclusion. Forty-six records were deemed eligible for full-text screening, from which fourteen new publications were included in the updated review. Six of the eighteen tools originally identified by Gazan *et al.* had new relevant publications since 2019. These fourteen publications described six studies on the Automated Self-Administered 24-h (ASA24) dietary assessment tool (*n* 2 validation^(10,11)^, *n* 3 usability^(12,13,14^^)^, *n* 1 both validation and usability^(15)^), two studies on Intake24 (*n* 2 usability^(16,17)^), two studies on Myfood24 (*n* 1 validation^(18)^, *n* 1 usability^(19)^), two studies on R24W (*n* 2 validation^(20,21)^), one study on NutriNet-Salud (*n* 1 development^(22)^) and one study on Foodbook24 (*n* 1 usability^(23)^). Data extracted on the development, validation and usability of each tool is not included in this short communication but can be provided upon request.

### Evaluation

#### Selection of shortlist

Table 2 shows the scores for each of the 24-h dietary recall tools. Based on the scoring of the pre-selection criteria, three of the eighteen tools were shortlisted: ASA24 (9/10), Intake24 (10/10) and Myfood24 (9/10).

**Table 2.** 24-h dietary recall tools scored against the pre-selection criteria

	Criteria #									
	1	2	3	4	5	Subtotal	6	7	8	Total
Max score	2	2	1	1	1	7	1	1	1	10
ASA24	2	1	1	1	1	6	1	1	1	9
CANAA-W	0	0	0	1	1	2				
CAPIS	0	0	0	0	1	1				
Clin Share	0	0	1	0	0	1				
Compleat™	1	1	1	0	0	3				
Crème Diet/Foodbook24	2	1	0	1	1	5				
Diet Advice	1	0	0	1	1	3				
Diet Day	1	0	0	1	1	3				
FBQ	1	1	0	1	1	4				
FoRC	1	0	0	1	1	3				
Intake24	2	2	1	1	1	7	1	1	1	10
Myfood24	2	1	1	1	1	6	1	1	1	9
NutriNet-Sante/NutriNet-Salud	1	2	1	0	1	5				
PAC24	1	0	0	0	1	2				
R24W	2	1	0	1	1	5				
RiksmatenFlex	1	2	0	0	1	4				
SACANA	1	2	0	1	1	5				
Web-SPAN	1	1	0	1	1	4				

ASA24, Automated Self-Administered 24-h dietary assessment tool; CANAA-W, Children’s and Adolescents’ Nutrition Assessment and Advice on the Web; CAPIS, Computer-Assisted Personal Interview System; FBQ, Web-based Food Behaviour Questionnaire; FoRC, Food Record Checklist; Myfood24, Measure Your Food on One Day; PAC24, Portuguese self-administered computerised 24-h dietary recall; R24W, web-based 24-h dietary recall; SACANA, Self-Administered Children, Adolescents, and Adult Nutrition Assessment; Web-SPAN, Web-Survey of Physical Activity and Nutrition.

The first five criteria were scored and subtotalled. Tools that did not meet criteria 3 or 4 or scored ≤ 4 for criteria 1–5 were eliminated. The remaining tools were scored for criteria 6–8.

Criteria: (1) evidence of validation of 24-h dietary recall tool; (2) evidence of use in previous (national) surveys or large studies; (3) the tool is available for use; (4) the tool is in English or can easily be adapted to English; (5) the tool can use images to portray portion size; (6) the tool can record (or can be easily adapted to record) additional details required to accurately code dietary data; (7) the tool can be updated to align with the expected requirements for a New Zealand national nutrition survey; and (8) the tool can automatically link food/drink consumption to food composition data.

#### Evaluation and consultation

The three shortlisted 24-h dietary recall tools were assessed against the evaluation criteria described above. Findings and key strengths and limitations were summarised (Table 3) and presented to expert advisory groups. Across the three tools, only a few differences were observed, including the tools’ offline use, use with children and costs associated with modifications, maintenance and research use. Expert advisors highlighted the need for offline use of the tool to allow for data collection in rural or remote communities in New Zealand where access to Wi-Fi and mobile data may be limited. Myfood24 has the option to collect data offline but only using an Android application, limiting the use in a national nutrition survey in which laptops are the most likely device used for data collection. Intake24 allows local installation of the tool on researchers’ laptops to enable offline data collection with the data synced to a server at a later stage. Contact with ASA24 confirmed that it is a web-based programme only and therefore not downloadable to be completed offline, with no future plans to develop this offline functionality.

**Table 3.** Overview of strengths and limitations for the shortlisted 24-h dietary recall tools

Tool	Strengths	Limitations
ASA24	• Uses a researcher website that lets researchers manage the survey and data that has security/confidentiality measures in place. • Tool is in English, and the language can be further adapted. • Tool is easy to use for participants with some issues reported that are not specifically related to ASA24 but more common to 24-h dietary recall tools in general. • Tool is compatible with common browsers and can be used on desktops, laptops, tablets and smartphones. • Takes on average 24 min to complete. • Tool used in large studies to track dietary intake over time. • Well-validated tool that uses a multiple-pass format including the option to add important prompts and checks for long-time gaps between meals. • No licence fee. • Can adapt food list and nutrient database. • Tool can record dietary supplement intake. • Tool can enter and save recipes. • Tool can include a follow-up survey URL. • Australian ASA24 available (older version compared with the USA).	• Mainly designed for and tested with people from the age of 10 years old and upwards. • High costs to adapt the food list and nutrient database and incorporate New Zealand-based respondent feedback reports. • Tool cannot be used offline and use as an interviewer-led tool might be limited. • Common user usability issues relate to difficulties with navigating website/logging in, finding the correct food and portion size estimation. • Does not include a pre-check for accuracy/quality based on overall energy intakes. • Does not include warnings for unusual values/over/under consumption. • Tool does not assess the usual salt intake.
Intake24	• Uses a researcher website that lets researchers manage the survey and data with the potential to install and provide the system locally. • Great flexibility in terms of adapting language, interface, food and nutrient databases and respondent feedback reports, including support from the Intake24 team. • Originally developed for people over the age of 10 years old, but adaptions were also made to use the tool from the age of 1·5 years (data not published). • Validated tool that uses a multiple-pass format (fewer studies conducted compared with ASA24) including the option to include important prompts, checks for low energy reports and long-time gaps between meals. • Takes on average 20 min to complete. • Tool is easy to use for participants with some issues reported that are not specifically related to Intake24 but more common to 24-h dietary recall tools in general (fewer studies conducted compared with ASA24). • Tool is compatible with common browsers and can be used on desktops, laptops, tablets and smartphones. • No licence fee. • Dietary intake data is available ongoing on the web platform. • Tool can record dietary supplement intake. • Tool can include a follow-up survey URL or an external online questionnaire that can embed a participant’s unique URL to link to Intake24. • Used in the UK, is currently being adapted for use in Australia in their next national nutrition survey, and a New Zealand version is available. • Tool has been adapted for offline use and could be used to enter recall data collected on paper. • Tool can be used effectively by interviewers.	• Tool does not include a participant request/reminder management system. This has to be provided separately. • Currently not able to use recipe function. • Costs associated with support for localisation/internationalisation. • Common user usability issues relate to difficulties with navigating website/logging in and finding the correct food. • Does not include a warning for unusual values/overconsumption. • Tool does not assess the usual salt intake (adaption could be considered).
Myfood24	• Uses a web interface for researchers to manage the survey. • Great flexibility in terms of adapting language, interface, food databases and respondent feedback reports including support from the Myfood24 team. • Originally developed for people over the age of 10 years old, and usability studies show appropriate for adolescents and older people. • Validated tool (against biomarkers) that uses a multiple-pass format including the option to include important prompts, forgotten foods at the end and warning for unusually large quantities. • Takes on average 15 min to complete. • Tool is mostly easy to use for participants. • Can be used on common browsers, accessed via a computer, laptop or mobile. • Can include dietary supplements (not included in nutrient data). • Tool includes a recipe builder function. • Can link to online questionnaires. • Used in large studies in the UK, an Australian version is available and a New Zealand baby/infant food database exists. • Can be completed offline (Android device only) and can be administered by an interviewer.	• Not validated with children under 10 years. • May be expensive as there is a standard pricing model, annual licence fee, plus a fee per anticipated use of the system for research use. • Some usability-related comments state that searching for a food is challenging, which may be due to the underlying food list database (lack of home-made foods, lack of specific brands, etc). • Only includes a few prompts and can only be added for an additional fee. • Foods can only be added to the food list database by the Myfood24 team and with a development fee. • Does not include warnings for underconsumption or unusual energy intake. • Tool does not assess the usual salt intake.

ASA24, the Automated Self-Administered 24-h dietary assessment tool.

Based on the best available information. Where needed, information was verified with authors/developers.

Given the nutrition survey will include participants from the age of 2 years, advisory group members preferred a tool that had been designed for and tested with a wide range of age groups, including younger children. All three shortlisted recall tools were created for use with people aged 10 years and older. However, evidence could only be found for adaptations to Intake24 to make it suitable for use with younger ages (1·5 years and over).

Modifications needed to create a tool suited for use in the New Zealand population would require substantial efforts and close collaboration for each shortlisted tool. Costs associated with this process, but also the maintenance and use of the dietary recall tools, however, varied significantly. Both ASA24 and Intake24 charge no licence fee, while Myfood24 has an annual licence fee and an additional fee per anticipated use of the system (e.g. based on the number of participants, recalls and time points). Furthermore, new foods and prompts can only be added by Myfood24, and additional charges apply. Adaptation of Myfood24 was therefore more restricted and costly compared with Intake24 and ASA24.

Feedback from the experts was used to recommend a preferred 24-h dietary recall tool. Representatives of the Ministries of Health and Primary Industries then reviewed the full evaluation report, including expert advice, and made a final decision on the tool to be used in a national nutrition survey. Intake24 was considered best suitable for use in a national nutrition survey for three main reasons: (1) it can be used offline; (2) it has been used or is being adapted for use in national surveys by countries similar to New Zealand, including Australia and the UK, thus providing opportunities to establish collaborations, learn from other user experiences and compare survey findings; and (3) Intake24 has been developed for and tested in both child and adult populations, which was a key requirement for a New Zealand national survey.

## Conclusion

The rigorous evaluation process outlined in this short communication enabled the selection of a robust 24-h dietary recall tool for use in a New Zealand nutrition survey. It facilitated efficient and detailed data collection to determine the key strengths and limitations of online tools including their potential to be modified for the unique population demographics in New Zealand. This approach could be used as a guide for other countries when selecting a new 24-h dietary recall tool for use in a national nutrition survey.
